# Transcriptional profiling and pathway analysis reveal differences in pituitary gland function, morphology, and vascularization in chickens genetically selected for high or low body weight

**DOI:** 10.1186/s12864-019-5670-9

**Published:** 2019-04-25

**Authors:** Laura E. Ellestad, Larry A. Cogburn, Jean Simon, Elisabeth Le Bihan-Duval, Samuel E. Aggrey, Mardi S. Byerly, Michel J. Duclos, Tom E. Porter

**Affiliations:** 1 Department of Poultry Science, University of Georgia, Athens, GA 30602 USA; 2 Department of Animal and Avian Sciences, University of Maryland, College Park, MD 20742 USA; 3 Department of Animal and Food Sciences, University of Delaware, Newark, DE 19716 USA; 4 Biologie des Oiseaux et Aviculture, Institut National de la Recherche Agronomique (INRA), Université de Tours, UR83 Recherches Avicoles, 37380 Nouzilly, France

**Keywords:** Adenohypophysis, Growth, Metabolism, Body composition, Abdominal fatness, Pituitary hormones, Secretion, Angiogenesis, Cellular compromise, Microarray analysis

## Abstract

**Background:**

Though intensive genetic selection has led to extraordinary advances in growth rate and feed efficiency in production of meat-type chickens, endocrine processes controlling these traits are still poorly understood. The anterior pituitary gland is a central component of the neuroendocrine system and plays a key role in regulating important physiological processes that directly impact broiler production efficiency, though how differences in pituitary gland function contribute to various growth and body composition phenotypes is not fully understood.

**Results:**

Global anterior pituitary gene expression was evaluated on post-hatch weeks 1, 3, 5, and 7 in male broiler chickens selected for high (HG) or low (LG) growth. Differentially expressed genes (DEGs) were analyzed with gene ontology categorization, self-organizing maps, gene interaction network determination, and upstream regulator identification to uncover novel pituitary genes and pathways contributing to differences in growth and body composition. A total of 263 genes were differentially expressed between HG and LG anterior pituitary glands (*P* ≤ 0.05 for genetic line-by-age interaction or main effect of line; ≥1.6-fold difference between lines), including genes encoding four anterior pituitary hormones. Genes involved in signal transduction, transcriptional regulation, and vesicle-mediated transport were differentially expressed and are predicted to influence expression and secretion of pituitary hormones. DEGs involved in immune regulation provide evidence that inflammation and response to cellular stressors may compromise pituitary function in LG birds, affecting their ability to adequately produce pituitary hormones. Many DEGs were also predicted to function in processes that regulate organ morphology and angiogenesis, suggesting pituitary gland structure differs between the divergently selected lines.

**Conclusions:**

The large number of DEGs within the anterior pituitary gland of birds selected for high or low body weight highlights the importance of this gland in regulating economically important traits such as growth and body composition in broiler chickens. Intracellular signaling, transcriptional regulation, and membrane trafficking are important cellular processes contributing to proper hormone production and secretion. The data also suggest that pituitary function is intimately tied to structure, and organization of the gland could influence hypothalamic and systemic metabolic inputs and delivery of hormones regulating growth and metabolism into peripheral circulation.

**Electronic supplementary material:**

The online version of this article (10.1186/s12864-019-5670-9) contains supplementary material, which is available to authorized users.

## Background

The hypothalamus and pituitary gland are central components of the neuroendocrine system, which integrates internal and external cues to regulate important physiological processes including growth, metabolism, response to stress, and reproduction. At the central level, the hypothalamus transmits central nervous system signals in the form of releasing factors and release-inhibiting factors to the anterior pituitary gland, which relays information to target endocrine organs through secretion of trophic hormones [1]. Three of the six anterior pituitary hormones, adrenocorticotropic hormone [ACTH; derived from proteolytic cleavage of pro-opiomelanocortin (POMC)], thyroid-stimulating hormone (TSH), and growth hormone (GH), are important regulators of growth, metabolism, and body composition in all vertebrates.

Primarily through genetic improvements aimed at enhancing growth and nutrient utilization, broiler (meat-type) chickens have emerged as a fast-growing and efficient high-quality protein source for human consumption. Despite the importance of understanding endocrine control of growth and metabolism in chickens as it relates to optimizing efficiency of feed utilization, these processes are still not clearly understood [2, 3]. Two valuable experimental genetic model systems of selection for high and low body weight are the high weight selected (HWS) and low weight (LWS) selected lines generated and maintained at Virginia Tech [4, 5], and the high growth (HG) and low growth (LG) lines of chickens developed and maintained at the Institut National de la Recherche Agronomique (INRA) [6]. The HWS and LWS lines have been continually selected for over 50 generations and now differ almost 10-fold in body weight at the age of selection (8 weeks) [4, 5, 7]. They have been widely used to investigate growth, metabolism, and feed intake in broiler chickens. Alterations in expression of components of the somatotropic axis, including minor differences in pituitary GH expression [8], and muscle regulatory genes [9] have been observed between HWS and LWS birds. Metabolic differences between these genetic groups are highlighted by alterations in pancreatic function, glucose homeostasis, and metabolic flux in skeletal muscle and adipose tissue [10–12]. The HG and LG lines of broiler chickens have been divergently selected for a large difference in body weight at two developmental stages (juveniles at 8 weeks and adults at 36 weeks) [6], and they differ markedly in their growth curves [13] and body composition, exhibiting more than a 3-fold difference in body weight [14, 15] and an almost 20-fold difference in abdominal fatness [15] by 6 to 7 weeks post-hatch. Heavier HG birds have increased muscle fiber number and size relative to LG birds [16, 17], and their muscle cells are more responsive to in vitro stimulation with insulin-like growth factor (IGF) 1 [18]. Despite this, muscle levels of IGF receptor do not appear to differ between the two lines [19]. Hepatic IGF1 and IGF2 mRNA, as well as circulating IGF1, IGF2, and insulin, are higher in HG birds between 1 and 6 weeks post-hatch [14]. Transcriptional profiling of abdominal fat in the two lines suggests that HG birds express higher levels of transcription factors linked to adipogenesis, while LG birds exhibit increased expression of transcripts for genes that promote energy expenditure and are involved in hemostasis [15]. In addition to these molecular and endocrine differences, several quantitative trait loci for growth [20], metabolism and body composition [21], and breast meat quality [22] have been identified through use of the HG and LG lines. The large difference in body weight and ancillary changes in body composition due to divergent selection make the HG and LG chickens an ideal model to investigate the genetic basis for extreme differences in growth and metabolism, two significant economic traits in this important agricultural species. Presently, we have used this population for the discovery of additional neuroendocrine factors that regulate growth and body composition.

Consumer demand for food produced in the absence of antibiotics continues to increase, providing particular challenges for poultry production systems in terms of maintaining bird health and growth efficiency [23]. Therefore, it is critical to understand fundamental biological processes that regulate growth and body composition of broiler chickens for successful development of novel alternatives to the use of antibiotic growth promoters. Uncovering genetic mechanisms that govern these processes in the anterior pituitary gland will provide much-needed information on factors regulating growth, feed efficiency, and nutrient utilization. In the present study, we used our Del-Mar 14 K Chicken Integrated Systems Microarray [24, 25] for transcriptional analysis of the anterior pituitary gland in HG and LG broiler chicken lines at 1, 3, 5, and 7 weeks post-hatch. Novel genes and gene interaction networks were identified that regulate expression and secretion of pituitary hormones and the structure and function of the anterior pituitary gland.

## Results

### Phenotypic characterization of HG and LG chickens

**Fig. 1:**
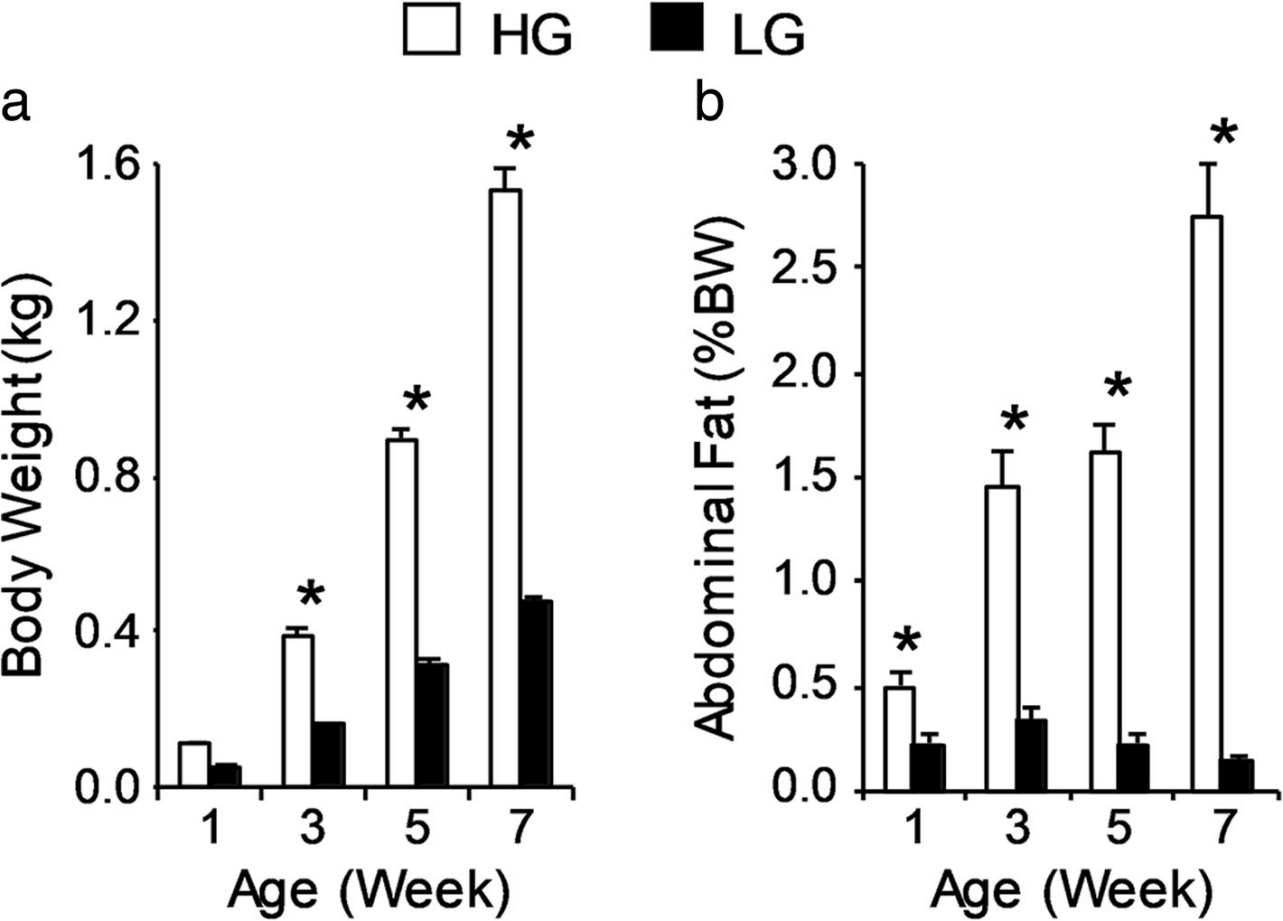
Phenotypic characterization of juvenile male HG and LG chickens. **a** Body weight (BW, kg; top) and **b** abdominal fat (% BW; bottom) of birds from each line at post-hatch weeks 1, 3, 5, and 7 (*n* = 8 birds per line). Values are expressed as mean ± SEM. When the two-way ANOVA demonstrated a significant line-by-age interaction (*P* ≤ 0.05), the presence of an asterisk (*) denotes a significant difference between the lines at the indicated age (*P* ≤ 0.05). Data included in this figure have been previously published in (21)

In order to assess phenotypic characteristics of birds used in this study (previously reported in [15]), body weights were determined and blood was collected for hormone analysis at time of tissue collection on post-hatch weeks 1, 3, 5, and 7. As shown in Fig. 1, body weight and percentage abdominal fat were significantly different between lines at most ages (*P* ≤ 0.05; *n* = 8). Divergence in body weight and abdominal fatness was most evident at week 7, where a 3.2-fold difference in body weight and a 19.6-fold difference in abdominal fat were observed. Thyroid hormones are primary regulators of longitudinal growth in birds. Plasma levels of the prohormone thyroxine (T_4_) exhibited a line-by-age interaction and were higher in HG birds than LG birds on week 7 (Table 1). Plasma levels of metabolically active 3,5,3′-triiodothyronine (T_3_) showed significant main effects of genetic line and age. Levels were elevated in the HG birds and decreased between weeks 1 and 7 in both lines (Table 1). Thus, LG chickens could be considered to be slightly hypothyroid when compared to the HG chickens.

**Table 1:** Plasma thyroxine (T_4_) and 3,5,3′-triiodothyronine (T_3_) levels in HG and LG chickens

	Week 1	Week 3	Week 5	Week 7	^b^*P*-values		
					Line x Age	Line	Age
^a^T_4_ (ng/ml)					0.0268	0.1154	0.0151
HG	24.9 ± 0.9	28.7 ± 1.2	27.9 ± 1.9	32.1 ± 0.5*			
LG	23.2 ± 1.4	29.9 ± 1.1	29.0 ± 2.2	24.2 ± 2.3*			
^a^T_3_ (ng/ml)					0.4427	0.0035	0.0027
HG	3.0 ± 0.2	2.7 ± 0.3	2.1 ± 0.1	2.2 ± 0.1			
LG	2.3 ± 0.2	2.2 ± 0.1	1.9 ± 0.2	1.7 ± 0.2			

^a^Values are mean ± SEM for *n* = 4 birds per line

^b^When the two-way ANOVA demonstrated a significant line-by-age interaction (*P* ≤ 0.05), the presence of an asterisk (*) denotes a significant difference between the lines at the indicated age (*P* ≤ 0.05)

### Expression profiles for pituitary hormones in HG and LG birds

**Fig. 2:**
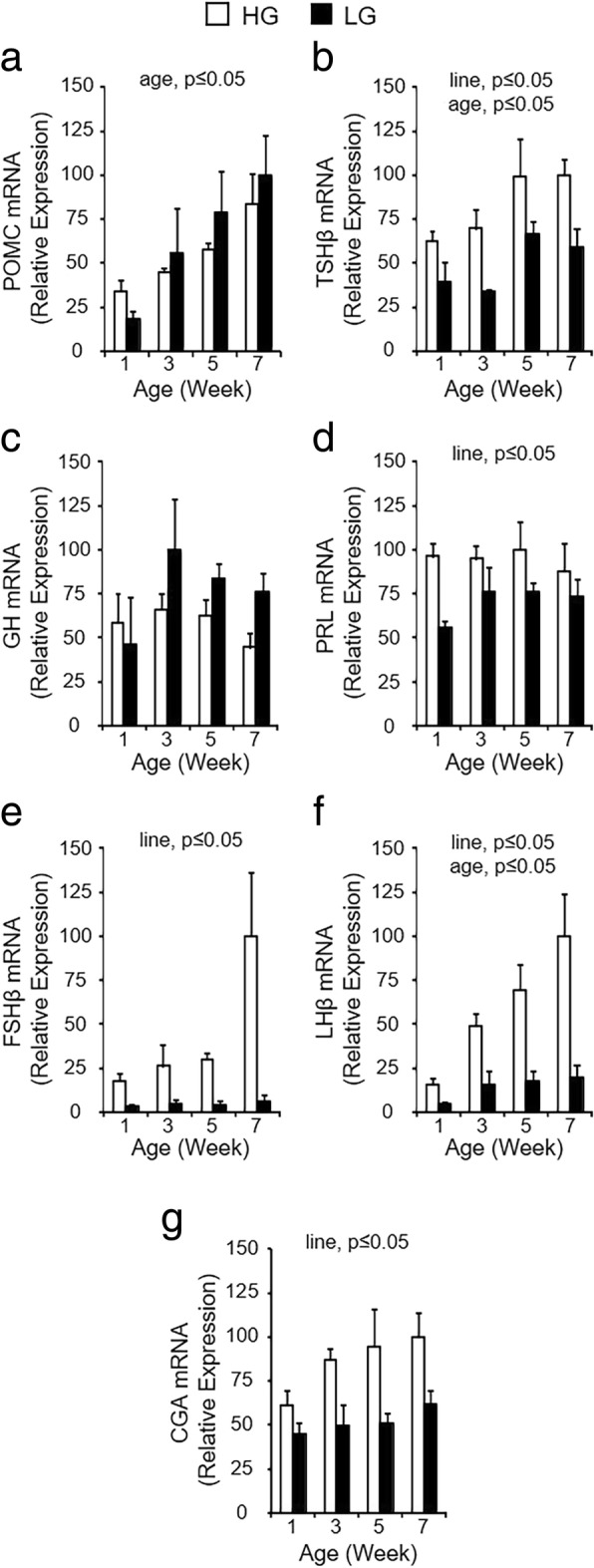
Gene expression profiles for pituitary hormones in HG and LG chickens. Levels of mRNA for POMC (**a**), TSHβ (**b**), GH (**c**), PRL (**d**), FSHβ (**e**), LHβ (**f**), and CGA (**g**) were determined on post-hatch weeks 1, 3, 5, and 7 and normalized to levels of GAPDH mRNA. Values (mean + SEM; *n* = 4 birds per line) are expressed relative to the age and line with the highest expression level (set to 100% prior to log_2_-transformation for statistical analysis). The line-by-age interaction was not significant for any hormone (*P* ≤ 0.05). Significant main effects of line and/or age (*P* ≤ 0.05) are indicated at the top of each graph, when present

The primary function of the anterior pituitary gland is synthesis and secretion of five major hormones: ACTH, TSH, GH, prolactin (PRL), follicle-stimulating hormone (FSH), and luteinizing hormone (LH). TSH, FSH, and LH are heterodimers consisting of a common α-glycoprotein hormone subunit (CGA) and distinct hormone-specific β-subunits. Differences in pituitary hormone expression between HG and LG birds were determined by reverse transcription-quantitative PCR (RT-qPCR) analysis (Fig. 2). There was a significant increase in expression levels of *POMC*, *TSHβ* and *LHβ* mRNA between post-hatch weeks 1 and 7, and a main effect of line where HG birds had higher levels of *TSHβ*, *PRL*, *FSHβ*, *LHβ*, and *CGA* mRNA than LG birds (*P* ≤ 0.05; *n* = 4). There were no significant differences in pituitary GH expression over time or between lines as determined by RT-qPCR (*P* > 0.05; n = 4), although birds in the LG line tended to have higher levels of pituitary *GH* mRNA on weeks 3, 5, and 7. These results indicate that normal synthesis, and potentially secretion, of a majority of the pituitary hormones are substantially compromised in LG birds.

### Transcriptional profiling of pituitary mRNA expression

In order to investigate differences in expression which could be contributing to altered pituitary hormone production and, ultimately, divergent growth and body composition, global gene expression profiling using the Del-Mar 14 K Chicken Integrated Systems Microarray was conducted in anterior pituitary glands of HG and LG broilers at weeks 1, 3, 5, and 7 post-hatch. A summary of the microarray data is given in Table 2. The array contains 19,200 spots representing 14,053 unique genes, over half of the known chicken genome. There were a total of 2312 cDNA probes that were significantly different across all comparisons. Of these, the genes of most interest were identified by the 1631 cDNA probes that were significantly different (*P* ≤ 0.05; *n* = 4) for the main effect of line (1100 probes) or the line-by-age interaction (353 probes). These effects indicate that expression is consistently higher or lower in one of the lines (main effect of line) or developmental expression profiles differ by line (line-by-age interaction). In our dataset, differentially expressed genes (DEGs) were defined as those that were detected in at least half the samples, had a statistically significant line or line-by-age effect (P ≤ 0.05), and a 1.6-fold difference between lines. Based on these criteria, there were 291 DEGs between the HG and LG pituitaries, and these represented 263 unique DEGs due to duplication of cDNA probes on the array. Of note, this dataset of DEGs included four pituitary hormones, namely *GH*, *TSHβ*, *LHβ*, and *FSHβ* (Additional file 1). To facilitate downstream functional analysis, these DEGs were annotated using the GeneBase tool on our website (http://cogburn.dbi.udel.edu/), which provides protein identifiers for cDNA microarray probes. Of the 291 DEGs submitted, 260 were successfully annotated with protein identifiers.

**Table 2:** Summary of microarray analysis

Category	Number
Spots printed on array	19,200
Spots with cDNA probes	17,834
Unique genes represented	14,053
^a^cDNA probes within trimmed dataset	10,437
^b^Significant cDNA probes	2310
Line-by-age interaction	353
Main effect of line	1100
Main effect of age	1058
^c^Differentially expressed genes	291
Line-by-age Interaction	110
Main effect of line	181
Main effect of age	72
^d^Genes annotated with GeneBase	260

^a^cDNA probes remaining after quality control check for removal of any that were undetectable, below background, malformed, had saturated pixel intensity, or were not present on at least half of the microarray slides

^b^cDNA probes with detectable background-corrected signal on at least 16 microarray slides and significantly different at *P* ≤ 0.05

^c^cDNA probes with detectable background-corrected signal on at least 16 microarray slides, *P* ≤ 0.05 for line-by-age interaction or main effect of line, and a difference between groups of 1.6-fold or greater (0.68-fold on a log_2_-scale)

^d^Number of 291 differentially expressed genes successfully annotated with protein identifiers using the GeneBase tool at http://cogburn.dbi.udel.edu/

**Fig. 3:**
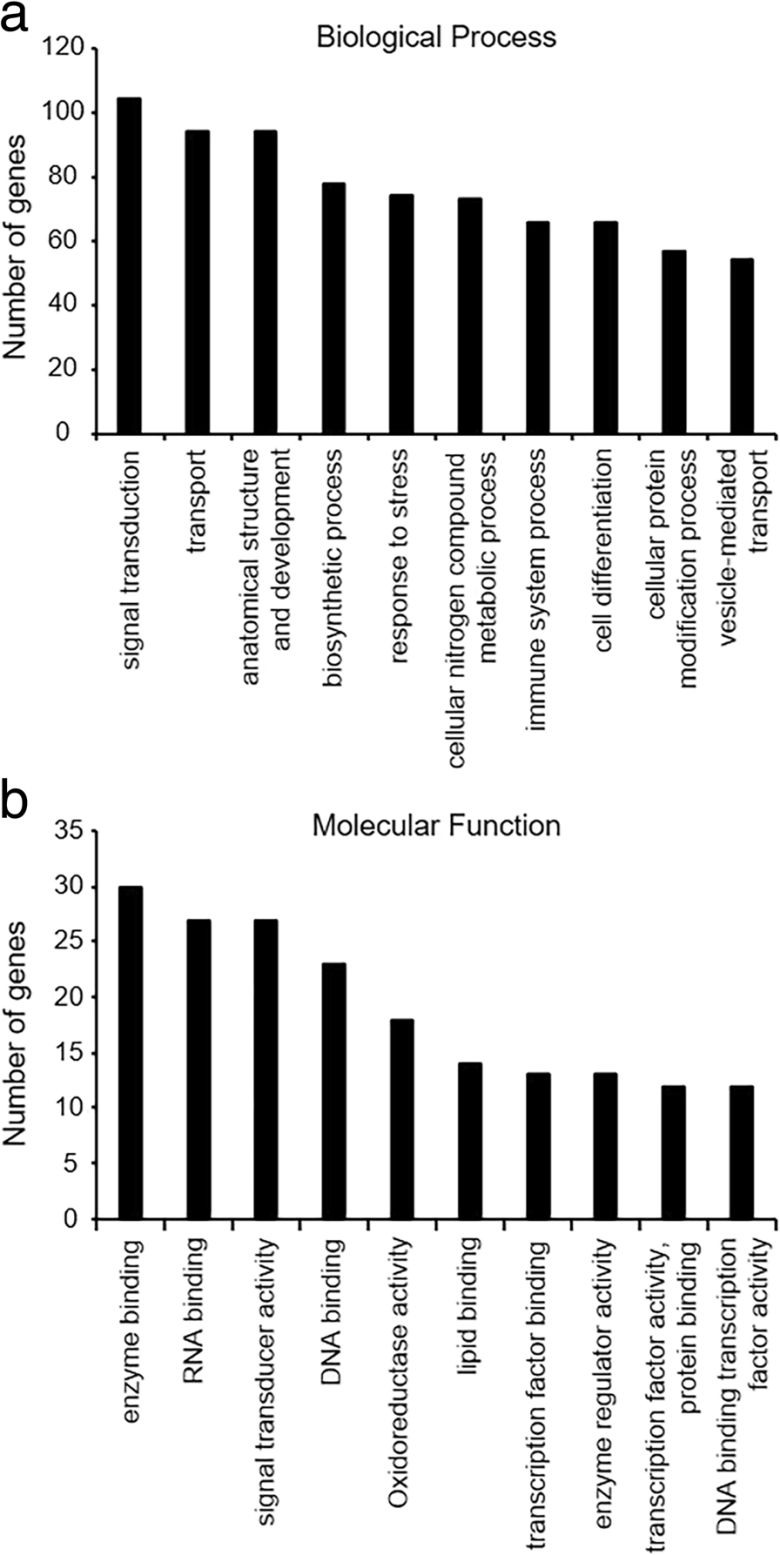
GO analysis of DEGs in the anterior pituitary gland of HG and LG chickens. Genes with a significant line-by-age interaction or a significant main effect of line (*P* < 0.05; *n* = 4 birds per line) as determined by two-way ANOVA and a difference in expression of at least 1.6-fold between HG and LG lines (291 total) were organized into GO categories based on biological process (**a**) and molecular function (**b**), using the GORetriever and GOSlimViewer tools on AgBase (http://agbase.arizona.edu/). Shown are results for the top 10 groups within each annotation category

DEGs were organized into gene ontology (GO) categories in order to identify cellular biological processes and molecular functions that could contribute to differences in growth and body composition between the genetically divergent lines (Fig. 3). For biological process, the largest number of genes were included in categories involved in signal transduction (104 total DEGs), transport (148 total DEGs), anatomical structure and development (94 total DEGs), and response to stress/immune system processes (140 total DEGs). Consistent with this, one of the top molecular functions identified was signal transducer activity (27 total DEGs), and a large number of genes were also categorized as playing a role in DNA binding or transcription (60 total DEGs). These results indicate that processes which are related to hormone expression and secretion, gland structure and morphology, and the immune response could lead to differences in pituitary function between the HG and LG lines.

**Fig. 4:**
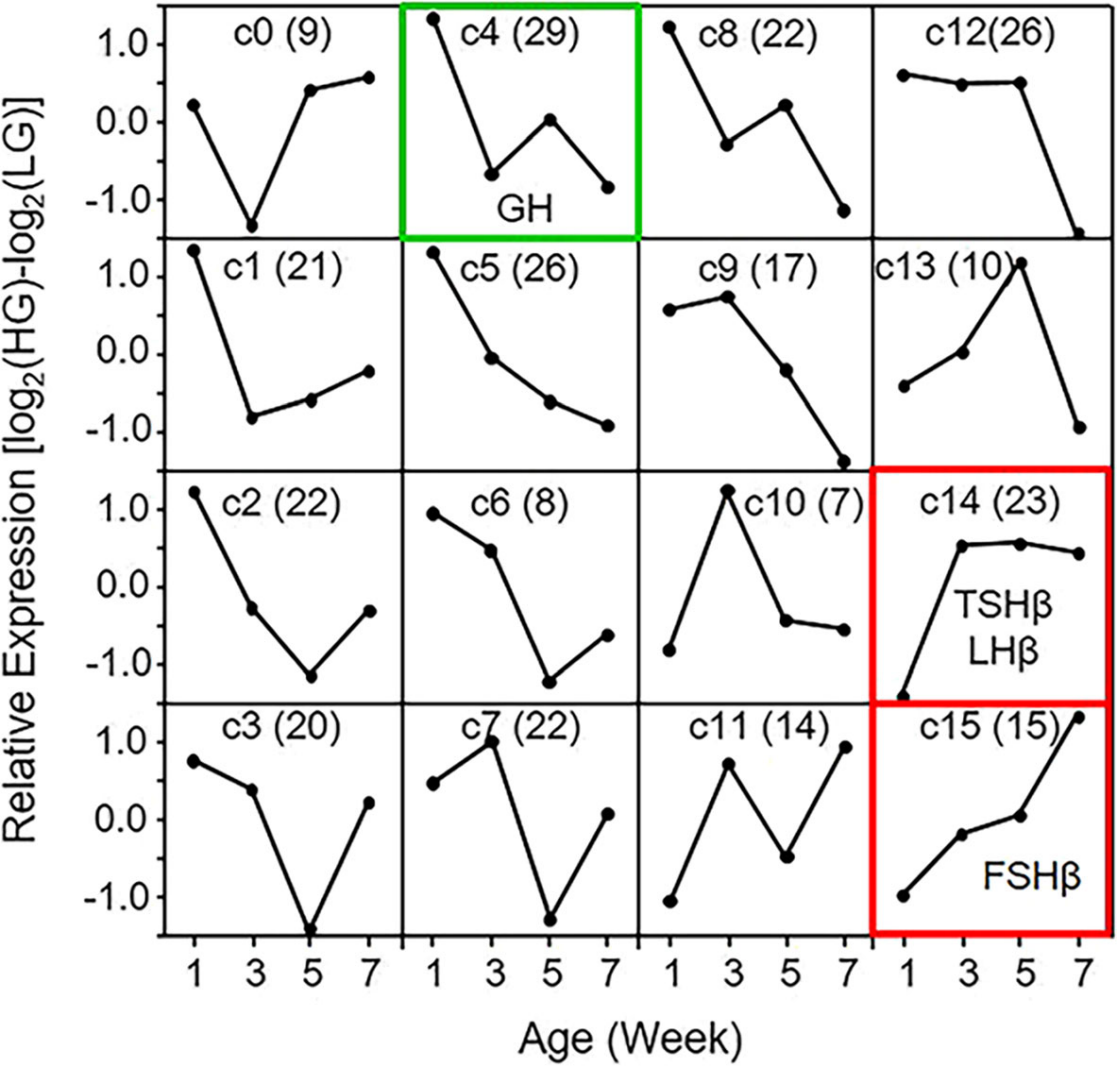
Self-organizing maps (SOMs) analysis of DEGs in the anterior pituitary of HG and LG chickens. Genes with a significant line-by-age interaction or a significant main effect of line (*P* ≤ 0.05; *n* = 4 birds per line) as determined by two-way ANOVA and a difference in expression of at least 1.6-fold between HG and LG lines (291 total) were organized into 16 clusters (c0-c15) in a 4 × 4 configuration. Data were organized into clusters as mean log_2_-ratio of HG – mean log_2_-ratio of LG. The number of genes contained within each cluster is indicated in parentheses at the top center of each cluster. Examples of important genes upregulated in LG chickens (green) or HG chickens (red) are identified in c4 (*GH*), c14 (*TSHβ and LHβ*) and c15 (*FSHβ*)

To identify alterations in pituitary gene expression between the lines that could affect growth and metabolism, DEGs were also subject to self-organizing maps (SOMs) analysis. For each of the 291 DEGs, relative expression data (log_2_ratio_HGmean_ – log_2_ratio_LGmean_) for each age were organized into 16 clusters (c0 – c15) in a 4 × 4 configuration (Fig. 4, Additional file 2). Genes contributing to differences in phenotype should be contained in clusters where relative expression between the lines changed in a manner consistent with divergence in growth and adiposity (i.e. primarily after week 1), namely c1 (21 DEGs), c2 (22 DEGs), c4 (29 DEGs), c5 (26 DEGs), c6 (8 DEGs), c8 (22 DEGs), c9 (17 DEGs), c14 (23 DEGs), and c15 (15 DEGs). The relative expression patterns contained within these clusters changed substantially between weeks 1 and 3, the time period when body weight diverged, and remained different for the remainder of the ages examined. Genes for pituitary hormones were contained within c4 (*GH*), c14 (*TSHβ* and *LHβ*), and c15 (*FSHβ*), reflecting an increase in *GH* expression in LG birds over time and the increase in *TSHβ*, *LHβ*, and *FSHβ* at later ages in HG birds. Also contained within these and closely-related clusters are genes such as the following: secreted factors semaphorin 3F (*SEMA3F*) (c9) and semaphorin 3C (*SEMA3C*) (c15); receptors serotonin receptor 1B (*5HTR1B*) and glutamate receptor, ionotropic, AMPA 1 (*GRIA1*) (c8), frizzled-4 (*FZD4*) and fms-related tyrosine kinase 1 (*FLT1*) (c14), and plexin D1 (*PLXND1)* (c15); intracellular adenylate cyclase 2 (*ADCY2*) and dexamethasone-induced ras-related protein 1 (*RASD1*) (c4), chromogranin B (*CHGB*) (c9), and mitogen-activated protein kinase kinase kinase 7 (*MAP 3 K7*) (c14); and transcription factors UNC homeobox (*UNCX*) (c4), nuclear receptor subfamily 0, group B, member 1 (*NR0B1*) (c8), and mothers against decapentaplegic homolog 1 (*SMAD1*) (c14).

**Fig. 5:**
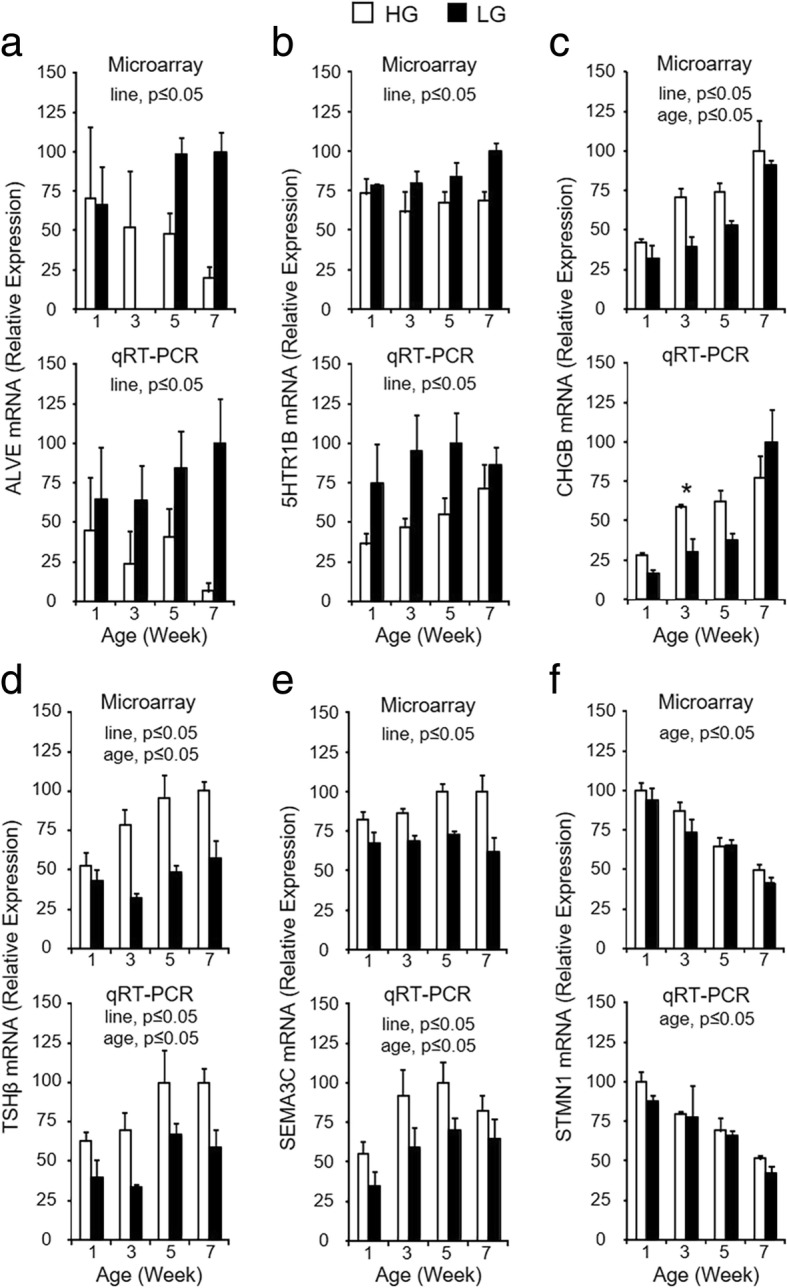
RT-qPCR confirmation of expression profiles determined by microarray and SOMs analysis for select DEGs. Microarray (**a**-**f**, top graph) and RT-qPCR (**a**-**f**, bottom graph) data are shown for each gene on post-hatch weeks 1, 3, 5, and 7. For RT-qPCR data, levels of mRNA for each gene were normalized to levels of GAPDH mRNA. Values (mean + SEM; *n* = 4 birds per line) are expressed relative to the age and line with the highest expression level for each technique (set to 100% prior to log_2_-transformation for statistical analysis). The RT-qPCR data for TSHβ mRNA (**d**, bottom) are also shown in Fig. 2b. When the two-way ANOVA demonstrated a significant line-by-age interaction (*P* ≤ 0.05), the presence of an asterisk (*) denotes a significant difference between the lines at the indicated age (*P* ≤ 0.05). Significant main effects of line and/or age (*P* ≤ 0.05) are indicated at the top of each graph when the line-by-age interaction was not significant (*P* > 0.05)

To confirm microarray expression patterns and selected relevant SOMs clusters, RT-qPCR was performed to measure mRNA expression for genes in five different clusters, as well as one that showed only a significant age effect (Fig. 5). Expression patterns of mRNA for avian leucosis virus envelope (*ALVE*) (c4), *5HTR1B* (c8), *CHGB* (c9), *TSHβ* (c14), *SEMA3C* (c15), and stathmin 1 (*STMN1*) (no cluster; age effect only) are comparable when determined by microarray (top graph in each panel) or RT-qPCR (bottom graph in each panel), confirming validity of the genome-wide transcriptional profiling and organization into SOMs clusters. Differences in expression between secreted factors and receptors, intracellular signaling molecules and chaperones, and transcription factors could affect pituitary function between HG and LG birds by contributing to differences in expression and secretion of pituitary hormones.

### Identification of gene interaction networks

**Fig. 6:**
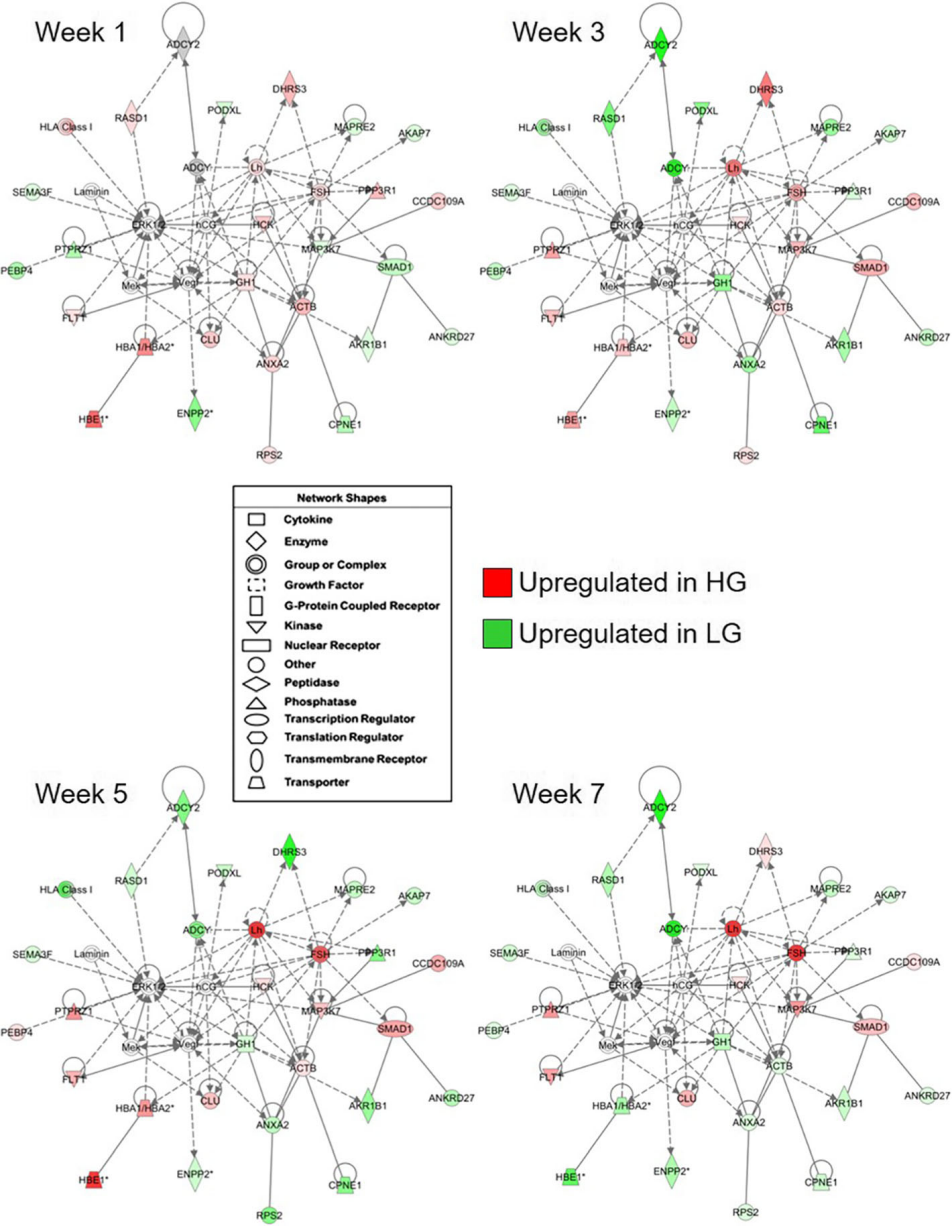
Functional gene interaction network associated with pituitary hormone production in HG and LG chickens. A gene network showing direct (solid lines) and indirect (dashed lines) relationships associated with “Molecular Transport” was identified using Ingenuity Pathway Analysis software. Genes which are colored red (upregulated in HG line) or green (upregulated in LG line) are contained within the dataset of 291 genes with a significant line-by-age interaction or a significant main effect of line (*P* ≤ 0.05; *n* = 4 birds per line) and a difference in expression of at least 1.6-fold between lines. The intensity of the red or green colored gene symbols indicates the magnitude of the difference between genetic lines. The legend indicates the functional type of each gene

**Fig. 7:**
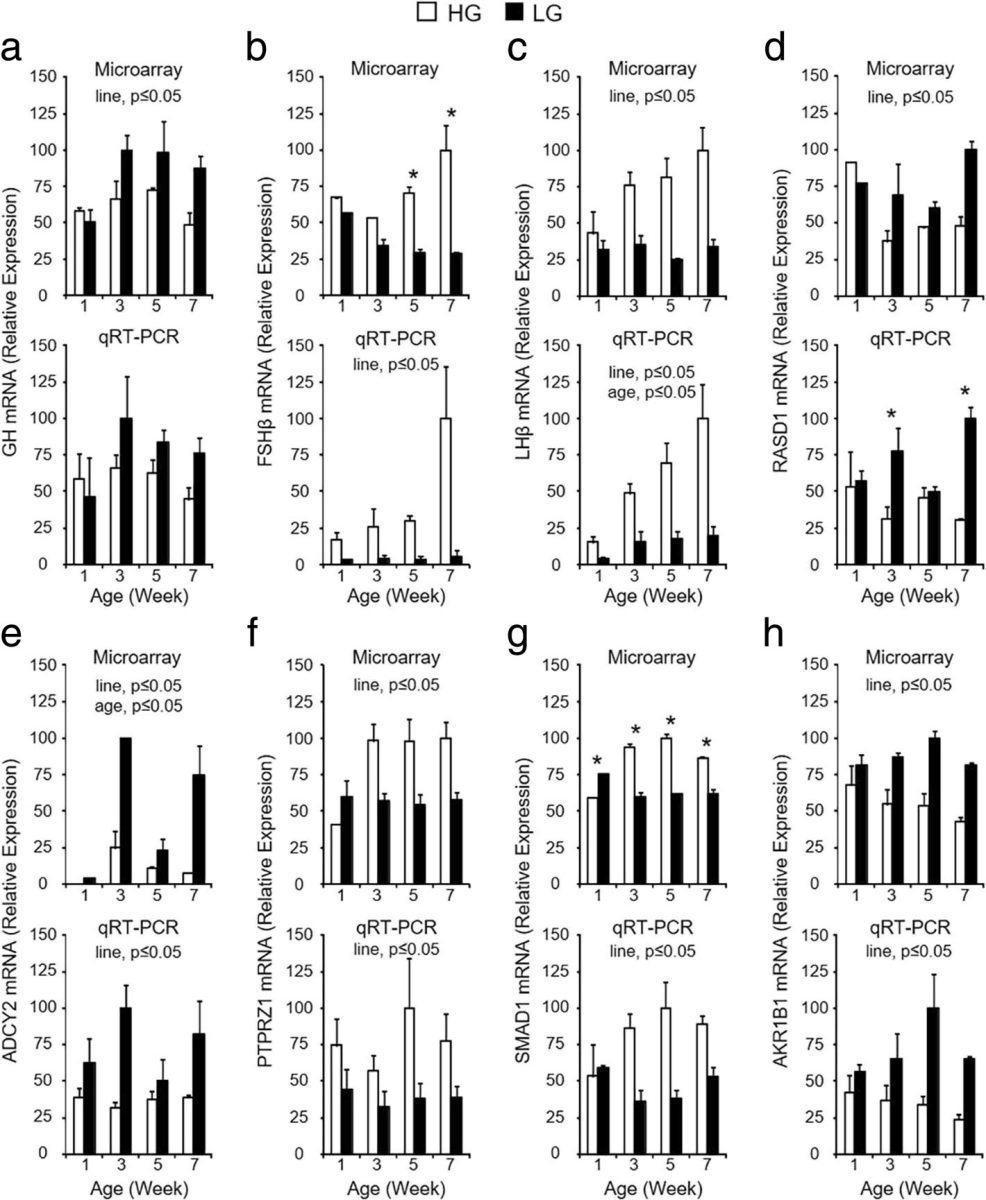
RT-qPCR confirmation of DEGs contained within the gene interaction network associated with pituitary hormone production. Microarray (**a** - **h**, top graph) and RT-qPCR (**a** - **h**, bottom graph) data are shown for each gene on post-hatch weeks 1, 3, 5, and 7. For RT-qPCR data, levels of mRNA for each gene were normalized to levels of GAPDH mRNA. Values (mean + SEM; *n* = 4 birds per line) are expressed relative to the age and line with the highest expression level for each technique (set to 100% prior to log2-transformation for statistical analysis). The RT-qPCR data for GH (**a**, bottom), FSHβ (**b**, bottom), and LHβ (**c**, bottom) are also shown in Fig. 2. When the two-way ANOVA demonstrated a significant line-by-age interaction (*P* ≤ 0.05), the presence of an asterisk (*) denotes a significant difference between the lines at the indicated age (*P* ≤ 0.05). Significant main effects of line and/or age (*P* ≤ 0.05) are indicated at the top of each graph when the line-by-age interaction was not significant (*P* > 0.05)

**Fig. 8:**
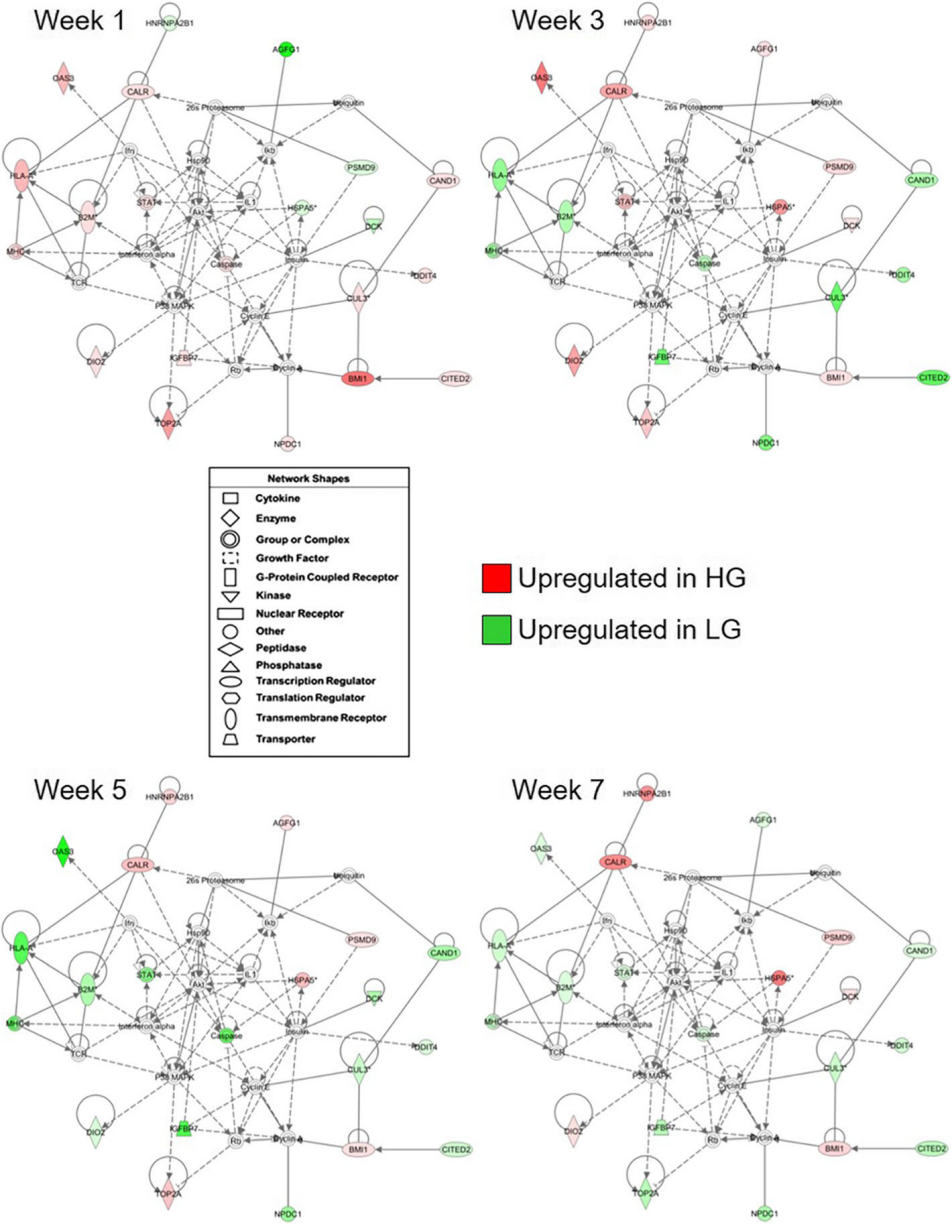
Functional gene interaction network associated with pituitary gland structure in HG and LG chickens. A gene network showing direct (solid lines) and indirect (dashed lines) relationships associated with “Organ Morphology and Cellular Compromise” was identified using Ingenuity Pathway Analysis software. Genes which are colored red (upregulated in HG line) or green (upregulated in LG line) are contained within the dataset of 291 genes with a significant line-by-age interaction or a significant main effect of line (*P* ≤ 0.05; *n* = 4 birds per line) and a difference in expression of at least 1.6-fold between lines. The intensity of the red or green colored gene symbols indicates the magnitude of the difference between genetic lines. The legend indicates the functional type of each gene

**Fig. 9:**
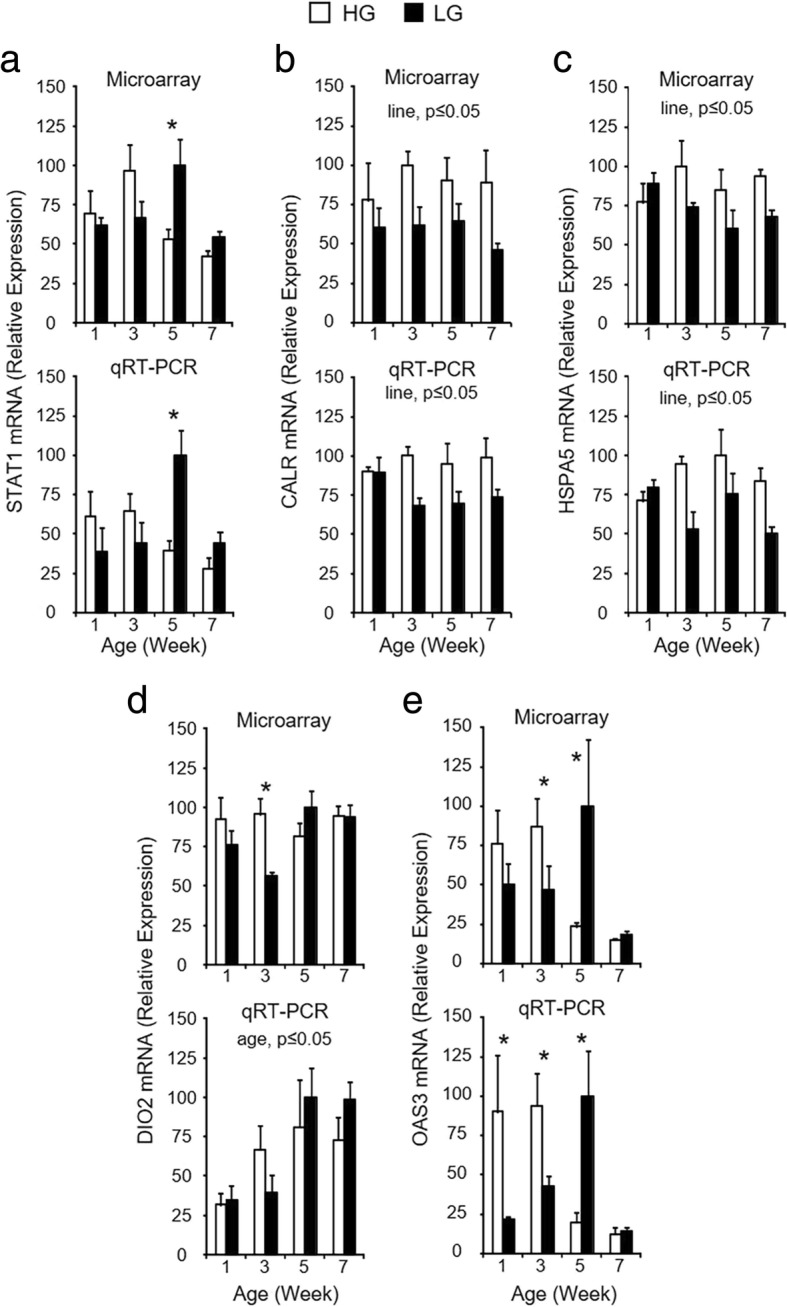
RT-qPCR confirmation of DEGs within the gene interaction network associated with pituitary gland structure. Microarray (**a** - **e**, top graph) and RT-qPCR (**a** - **e**, bottom graph) data are shown for each gene on post-hatch weeks 1, 3, 5, and 7. For RT-qPCR data, levels of mRNA for each gene were normalized to levels of GAPDH mRNA. Values (mean + SEM; *n* = 4 birds per line) are expressed relative to the age and line with the highest expression level for each technique (set to 100% prior to log_2_-transformation for statistical analysis). When the two-way ANOVA demonstrated a significant line-by-age interaction (*P* ≤ 0.05), the presence of an asterisk (*) denotes a significant difference between the genetic lines at the indicated age (*P* ≤ 0.05). Significant main effects of genetic line and/or age (*P* ≤ 0.05) are indicated at the top of each graph when the line-by-age interaction was not significant (*P* > 0.05)

To identify important pathways and gene networks associated with differences in pituitary mRNA expression between birds with different genetic growth potential, the functionally annotated gene list was submitted to Ingenuity Pathway Analysis (IPA) software for further functional annotation and analysis. Of the initial 260 DEGs, 249 had identifiers that mapped to the IPA annotated database and were eligible for incorporation into gene interaction networks and use in biological function identification. The top five gene interaction networks identified by IPA are listed in Table 3. The top network was associated with “Molecular Transport” (Fig. 6), and differential expression patterns of several genes within each network were confirmed by RT-qPCR (Fig. 7). A second network was identified as being associated with “Organ Morphology and Cellular Compromise” (Fig. 8), and RT-qPCR was used to confirm expression patterns of genes in this network (Fig. 9). Of particular interest are DEGs whose relative expression switches between weeks 1 and 3 or those whose magnitude increases over time, as they likely drive differences in phenotype.

**Table 3:** Top gene interaction networks containing DEGs within pituitary glands of HG and LG chickens

^a^Associated Network Functions	^b^ DEGs (#)	^c^Total Genes (#)
Molecular transport, inflammatory disease	30	34
Cancer, developmental disorder, lipid metabolism	29	33
Organ morphology, cellular compromise, cardiovascular system development and function	21	34
Endocrine system development and function, carbohydrate metabolism, small molecule biochemistry	16	34
Cell-to-cell signaling and interaction, cellular function and maintenance, tumor morphology	16	34

^a^As determined by Ingenuity Pathway Analysis gene interaction network prediction

^b^The number of DEGs between HG and LG birds from our dataset contained within each network

^c^The total number of genes contained within each network

The top network included genes involved in molecular transport (Fig. 6). This network contained several genes that play a role in signal transduction, including secreted ligands [vascular endothelial growth factor (*VEGF*) and *SEMA3F*], receptors [*FLT1* and protein tyrosine phosphatase, receptor type Z1 (*PTPRZ1*)], and intracellular signaling molecules [*RASD1*, *ADCY2*, hematopoietic cell kinase (*HCK*), *MAP3K7*, mitogen-activated protein kinase/extracellular signal-regulated kinase kinase 1 (*MEK1*), and extracellular signal-regulated kinase 1/2 (*ERK1/2*)] and regulatory subunits [protein phosphatase 3 regulatory subunit B alpha (*PPP3R1*) and A-kinase anchoring protein 7 (*AKAP7*)]. Also within this network and the dataset as a whole are genes for several calcium binding proteins involved in intracellular vesicular trafficking and protein synthesis quality control [annexin A2 (*ANXA2*), copine 1 (*CPNE1*), calreticulin (*CALR*), and heat shock protein family A, member 5 (*HSPA5*)], as well as membrane trafficking [ankyrin repeat domain-containing protein 27 (*ANKRD27*) and microtubule associated protein RP/EB family member 2 (*MAPRE2*). Three of the pituitary hormones (*GH*, *LH*, and *FSH*) in our dataset were also included, and differences in their expression levels between lines either switches or increases in intensity over time. This indicates that alterations in cellular signaling, membrane trafficking components, and vesicular secretion may contribute to differences in hormone expression and secretion between HG and LG birds. Interestingly, the network was also populated with genes with roles in angiogenesis [(*FLT1*, *VEGF*, *HCK1*, podocalyxin-like protein 1 (*PODXL*), and hemoglobin alpha 1/hemoglobin alpha 2 (*HBA1/HBA2*)], suggesting that alterations in microvasculature formation and support differ between HG and LG birds.

A second network of interest contained genes with a role in cellular compromise (Fig. 7). Within this network are genes associated with antigen presentation [beta-2-microglobulin (*B2M*)*,* major histocompatibility complex (*MHC*), human leukocyte antigen A (*HLA-A*), and T-cell receptor (*TCR*)], antiviral response [2′-5′-oligoadenylate synthetase 3 (*OAS3*)], and cytokine signaling or transcriptional regulation [interleukin-1, interferon alpha, inhibitor of kappa B, p38 mitogen-activated protein kinase*,* and signal transducer and activator of transcription 1 (*STAT1*)]. Genes which may be responsive to other cellular stressors were also included, such as those involved in apoptosis [caspase 8 (*CASP8*), cyclin E, protein kinase B/Akt, and retinoblastoma protein] or cellular responses to DNA damage [DNA damage-inducible transcript 4 (*DDIT4*)]. Comparing this network on post-hatch week 1 with those of later ages, it is clear by the increase in numbers and intensity of green-colored DEGs that after divergence in growth, LG birds have increased expression of factors associated with the immune response, inflammation, and other cellular stressors. Altogether, this indicates that gene expression patterns related to the immune response and other compromising events may contribute to differences in pituitary function.

The top biological functions associated with DEGs in the anterior pituitary of HG and LG birds are summarized in Table 4; DEGs contained within each category are listed in Additional file 3. In terms of molecular and cellular function, 65 DEGs were associated with cellular growth and proliferation or cell cycle, and 52 DEGs were associated with cell death or cellular compromise. A large number were also associated with cellular signaling. Physiological system development and function categories were heavily populated with genes involved in vasculature formation, including hematological and cardiac system development and function, and tissue and organ morphology. These biological functions further emphasize that differences in signaling, cellular proliferation, microvasculature formation, and morphology all affect pituitary gland function in HG and LG birds.

**Table 4:** Top biological functions of DEGs within pituitary glands of HG and LG chickens

^a^Top Biological Function	^b^DEGs (#)	^c^*P*-Value
Molecular and cellular function		
Cell death	46	9.96E-05 – 2.25E-02
Cell cycle	19	1.27E-04 – 2.25E-02
Cell-to-cell signaling and interaction	26	6.86E-04 – 2.25E-02
Cellular compromise	6	3.87E-04 – 2.09E-02
Cellular growth and proliferation	46	1.24E-03 – 2.20E-02
Physiological system development and function		
Cardiovascular system development and function	16	5.36E-05 – 2.25E-02
Organ morphology	12	5.36E-05 – 1.13E-02
Hematological system development and function	32	1.16E-04 – 2.05E-02
Tissue morphology	24	1.16E-04 – 2.05E-02
Endocrine system development and function	6	7.13E-04 – 2.25E-02

^a^As determined by Ingenuity Pathway Analysis (IPA) biological function prediction

^b^The number of differentially expressed genes between HG and LG birds from our dataset contained within each category

^c^*P*-values were determined by IPA software based on the number of DEGs from the dataset within each biological category divided by the total number of known genes assigned to that category within the database

### Upstream regulator analysis

**Fig. 10:**
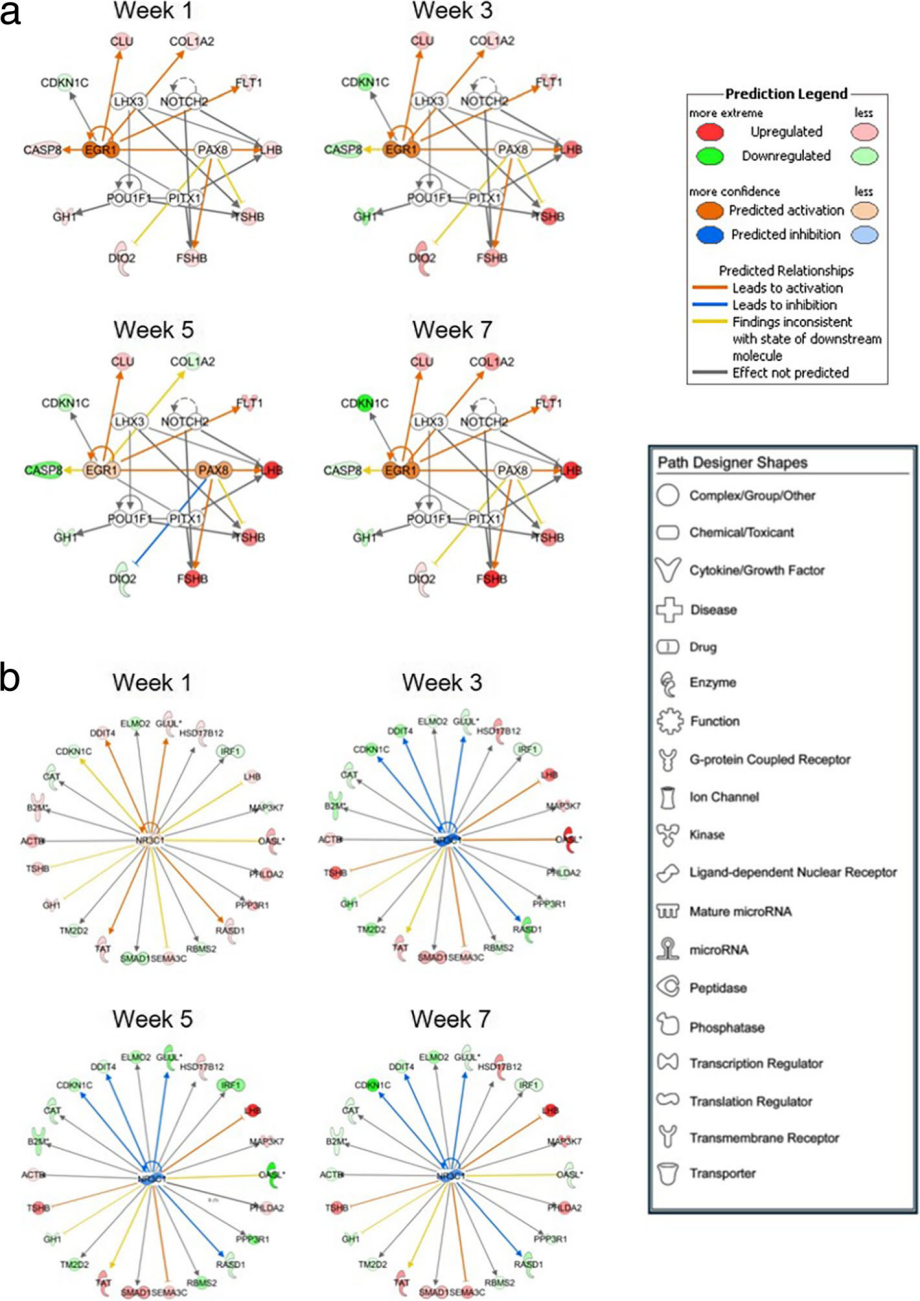
Transcriptional regulators predicted to regulate DEGs observed in the pituitary of HG and LG chickens. Upstream regulator analysis using Ingenuity Pathway Analysis was performed on the dataset of 291 genes with a significant line-by-age interaction or a significant main effect of line (*P* ≤ 0.05; *n* = 4 birds per line) and a difference in expression of at least 1.6-fold between lines. This analysis identified transcription factors with direct actions on differentially expressed target genes. **a** Transcriptional regulators associated with pituitary development and function that could contribute to differential expression of pituitary hormones were identified. **b** A large number of differentially expressed genes between the two lines are also predicted to be regulated by glucocorticoid receptor (*NR3C1*). Upstream regulators colored orange are predicted to have higher activity in HG birds, and upstream regulators colored blue are predicted to have higher activity in LG birds. Genes within the dataset that are colored red were more highly expressed in the HG line, and genes within the dataset that are colored green were more highly expressed in the LG line. The color of the line connecting transcriptional regulators to differentially expressed genes indicates IPA prediction of whether transcription factor activity should lead to upregulation (orange line) or downregulation (blue line) of the DEG targets

Using IPA, upstream regulator analysis was performed to identify transcription factors with direct actions on DEGs in the pituitary glands of HG and LG birds. Transcriptional regulators associated with pituitary development and function that potentially contribute to differential expression of pituitary hormones are shown in Fig. 10a. Predicted upregulation of paired box gene 8 (*PAX8*) and early growth response protein 1 (*EGR1*) in HG birds is likely responsible for differential expression of genes associated with cell turnover that could change pituitary structure and morphology [collagen, type I, alpha 2 (*COL1A2*), clusterin (*CLU*), cyclin dependent kinase inhibitor 1C (*CDKN1C*), *CASP8*], as well as microvascular formation (*FLT1*). Other transcription factors identified as upstream regulators in this network influence pituitary cell-type differentiation and hormone expression, indicating that these could also be differentially expressed between HG and LG birds. A second upstream regulator identified by IPA was nuclear receptor subfamily 3, group C, member 1 (*NR3C1*) or glucocorticoid receptor, which is predicted to be upregulated in HG birds on week 1 and in LG birds at later ages (Fig. 10b). LG birds exhibit a substantial increase in expression of genes associated with immunity, inflammation, and other cellular stressors after week 1, such as *B2M*, *DDIT4*, interferon regulatory factor 1 (*IRF1*), 2′-5′-oligoadenylate synthetase like (*OASL*), and *PPP3R1*. This may be a result of increased pituitary glucocorticoid receptor activity in these birds as compared to those in the HG line on weeks 3, 5, and 7.

## Discussion

Transcriptional profiling of anterior pituitary gene expression during the juvenile growth period of broilers selected for high or low body weight has confirmed an important role for this gland in regulation of growth and metabolism in chickens. A total of 363 DEGs were identified as being developmentally regulated with age in both lines (72 DEGs) or potentially contributing to phenotypic differences between HG and LG birds (291 DEGs). Of most interest are those genes in the latter category that exhibit overall line or line-by-age interactive effects, and gene expression profiles for 19 of these DEGs were confirmed by RT-qPCR. Several approaches were taken to analyze transcriptional profiling data in order to determine how these DEGs may contribute to alterations in pituitary function and, ultimately, the divergent growth and compositional phenotypes.

Differences in pituitary hormone expression between the lines indicate that LG birds exhibit compromised pituitary function that could be manifest in altered production and secretion of several important pituitary hormones. Reduced levels of pituitary *TSH* mRNA in LG birds are consistent with our observation that circulating thyroid hormones are lower in these birds and findings of others that T_3_, and to a lesser extent T_4_, positively regulate overall body growth in chickens [26, 27] and may contribute to increased abdominal fat in HG birds [28]. Differences in expression of transcripts known to be regulated by thyroid hormones (e.g. thyroid hormone responsive spot 14 alpha (*THRSPA*), as well as enzymes responsible for activation and inactivation of T_3_ (e.g. deiodinase (*DIO*) 1 and *DIO3*), were observed in adipose tissue from these birds [15], demonstrating that alterations in pituitary TSH production are driving differential thyrotropic axis activity that directly contributes to divergence in growth and adiposity. Pituitary mRNA levels of *DIO2*, another enzyme responsible for local conversion of T_4_ to T_3_, were different between the lines. This indicates that, in addition to differences in circulating levels of thyroid hormones, there is also a potential difference between the lines related to local thyroid hormone action at the pituitary gland that could influence production of hormones such as TSH and GH. Despite an apparent increase in the number of pituitary somatotrophs in fast-growing lines of birds during late embryonic development [29], it is well established that circulating GH is elevated in slower-growing lines of chickens after hatch [30, 31], and differences observed in pituitary *GH* mRNA levels in the current study were consistent with this. Pituitary *GH* mRNA was also shown to be higher in lighter birds 4 weeks after hatch using a similar genetic model system in which HWS birds weigh approximately 10-fold more than LWS birds at 8 weeks of age [8]. It has been demonstrated in different birds from these same lines that circulating GH levels were 2.5-fold higher in LG chickens than HG chickens (unpublished observation by LAC, TEP, JS, and MJD). Despite increased pituitary GH, LG birds exhibit reduced levels of both hepatic IGF1 and IGF2 mRNA as well circulating IGFs [14]. This suggests that the slower-growing LG birds may be deficient in the hepatic response to GH, which may be a result of decreased expression of GH receptor (GHR) in liver or disruption of intracellular GHR signaling. In fact, hepatic GH-binding in LG chickens was only one-seventh that of the HG birds between 5 and 11 weeks post-hatch (unpublished observation by LAC, JS, and MJD). These findings are similar to those reported earlier in sex-link dwarf (*dw/dw*) chickens, which have elevated plasma GH, no detectable hepatic GH-binding activity, and maintain two-thirds of the plasma IGF-1 levels despite lacking a functional GHR gene [32–35]. In contrast, hepatic *GHR* mRNA expression was observed to be higher early post-hatch in LWS chickens and only modestly reduced at 4 weeks of age when compared to HWS chickens, and it was reported that *GHR* mRNA levels in breast muscle are higher in LWS birds during both embryogenesis and post-hatch [8]. Others have found that breast muscle cells from LG birds exhibit reduced sensitivity to IGF1 stimulation [18], and it has been suggested that their visceral fat may have a reduction of IGF1 signaling when compared to HG birds [15]. Taken together, it is apparent that differences in pituitary hormone production and downstream actions of these hormones play a major role in altering metabolic phenotypes in these birds.

Differential expression of receptors and intracellular signaling molecules may contribute to differences in expression of pituitary hormones between HG and LG birds. Midkine (MDK) is a secreted protein produced by folliculostellate cells within the embryonic rat pituitary gland [36], and its receptor, *PTPRZ1*, was more highly expressed in HG birds after divergence in growth and body composition. *PTPRZ1* expression has recently been detected in the adult rat anterior pituitary [37], specifically within ACTH- and GH-producing cells, and the authors speculate that it mediates paracrine MDK signaling within these cell types. It is possible that MDK-PTRPZ signaling may be a novel regulator of these hormones, and differences in *PTPRZ1* expression between HG and LG birds may result in altered pituitary GH production. Many pituitary hormone releasing and release-inhibiting factors secreted by the hypothalamus activate G protein coupled receptors, which signal through generation of second messengers such as cyclic adenosine monophosphate (cAMP) and calcium, or MAPK pathways [38–40]. RASD1 has been shown to interfere with cAMP-stimulated peptide hormone secretion in a corticotroph cell line [41], and elevated *RASD1* mRNA levels in LG birds suggests that they have increased RASD1 activity that may contribute to the reduced expression of *TSHβ, FSHβ,* and *LHβ* mRNA in a similar manner. An increase in *ADCY2* expression in these birds may increase intracellular cAMP in an attempt to maintain hormone expression levels in the face of this interference. Further supporting differences in cAMP-mediated intracellular signaling between the lines is the observation that LG birds exhibit elevated levels of *AKAP7* mRNA, a scaffolding protein that binds to regulatory subunits of cAMP-activated protein kinase A and influences its activity [42]. Increased expression of mRNA for a regulatory subunit of calcineurin, *PPP3R1,* in LG birds indicates that calcium-mediated intracellular signaling may also be altered between the lines and influence pituitary hormone expression levels and/or secretion. Activin-mediated induction of FSH production is regulated by MAP3K7 in sheep pituitary cells [43], and elevated levels of *MAP3K7* mRNA in HG birds suggests that this pathway may play a similar role in birds and also be a positive regulator of pituitary hormones. In addition to differential cell signaling, HG birds exhibited higher levels of mRNA for genes associated with the basic cellular functions of transcription and translation, such as those involved in processing nascent mRNA transcripts [heterogeneous nuclear ribonucleoprotein A2/B1 (*HNRNPA2B1*)] [44], and chaperones involved in the misfolded protein response (*CALR* and *HSPA5*) [45]. Alterations in intracellular signaling within pituitary glands of HG and LG birds, as well as the ability to process newly transcribed and translated genes, likely leads to differences in pituitary hormone production between the lines.

In addition to differences in pituitary hormone expression, it is clear that hormone secretion may also be altered, as HG and LG birds exhibit differential expression of several genes with roles in molecular transport and calcium-dependent membrane trafficking. The *CHGB* gene is one of the most abundantly transcribed genes in the pituitary gland [46] and is a major component of dense-core secretory vesicles within endocrine, neuronal, and other secretory cell types [47]. The observation that *CHGB* expression was elevated in the anterior pituitary of HG birds indicates that the formation of secretory vesicles containing pituitary hormones may be compromised in birds from the LG line. Vesicular transport, fluctuation in intracellular calcium levels, and membrane fusion events are necessary for proper hormone secretion from these dense-core vesicles, and differences in expression levels for genes likely involved in these processes are evident between HG and LG birds. ANKRD27 is thought to interfere with vesicle-associated membrane protein 7-mediated vesicular fusion by trapping it in a conformation unable to interact with snap receptor proteins on the opposing membrane [48]. This interaction is crucial for proper membrane fusion and release of vesicular contents at the plasma membrane, and increased levels of *ANKRD27* mRNA indicate that this process may be less efficient and interfere with hormone secretion in LG birds. In mammalian cells, CPNE1, MAPRE2, an ANXA2 are thought to play a role in proper calcium-induced trafficking to and docking with the plasma membrane [47, 49, 50]. Upregulation of mRNA expression for these genes was observed in LG pituitary glands and may be a compensation for reduced synthesis and/or functionality of hormone-secreting vesicles in these birds.

Genes with a role in antigen presentation (*B2M*, *MHC*, and *HLA-A*) [51], the antiviral response (*OAS3*) [52], cytokine signaling and transcriptional regulation (*STAT1*) [53], apoptosis (*CASP8*) [54], and response to cellular stressors such as hypoxia and DNA damage response (*DDIT4*) [55] were elevated at later ages in the pituitary of LG birds. This may lead to complications similar to that seen in lymphocytic hypophysitis, which is characterized by immune cell infiltration, inflammation, and damage to pituitary cells that can result in differing levels of hypopituitarism [56–58]. Interestingly, in addition to its role in response to cellular stressors, DDIT4 has recently been implicated in a wide range of cellular processes which impact energy homeostasis and metabolic function [55]. Of note is that many of these genes are upregulated in LG birds after week 3, when the metabolic and growth phenotypes diverge. Similarly, after 3 weeks of age, pituitary glucocorticoid receptor activity is predicted to be upregulated in LG birds. This may be a response to alterations in expression of genes associated with cellular stressors and further interfere with normal pituitary function. Elevated pituitary glucocorticoid receptor activity in LG birds suggests that these birds may have higher levels of circulating corticosterone, leading to metabolic alterations that negatively impact growth.

Results from several studies have demonstrated that *ALVE* transcripts are overexpressed in several tissues from slower growing chickens at most ages examined [15, 59–62]. In the present study, *ALVE* mRNA expression was higher in LG chickens from week 3 onwards, as determined by both global transcriptional profiling and RT-qPCR (Fig. 8). In fact, it was one of the most highly DEGs between the genetic lines. Transcriptional analysis of important somatic metabolic tissues such as abdominal fat, liver, and breast muscle has also revealed that *ALVE* expression is highly upregulated in LG chickens [15, 59]. In a second, independently selected genetic model system, slower growing LWS chickens were observed to exhibit higher levels of *ALVE* mRNA in the hypothalamus, whole brain, liver, breast muscle, and adipose tissue [60–62]. These authors determined that more ALVE integration sites were detected in LWS than HWS chickens and contributed to differences in expression between the lines [62]. It is likely a similar phenomenon has occurred in our genetically divergent LG versus HG birds. While it is not known if this differential expression is a direct response to selection for high or low growth (i.e. whether the *ALVE* sequences are directly affecting growth or are linked to loci regulating growth), it is clear that presence and expression of the *ALVE* allele are highly correlated with reduced growth. In addition to the above possibilities, multiple integrations of *ALVE* may disrupt key functional genes that control growth and metabolism in chickens.

Several categories of differentially expressed genes indicate that it is likely there are differences in structure and organization within the neuroendocrine system of HG and LG broiler chickens. The homeobox transcription factor UNCX is involved in developing neuronal contacts between the hypothalamus and pituitary [63] and was differentially expressed between the lines, providing evidence that this connectivity may differ in HG and LG birds. Semaphorins are a class of secreted and membrane-tethered molecules that were first identified for their role in axon guidance, and plexins act as their cell-surface receptors [64]. Semaphorin-plexin D1 signaling has been shown to mediate VEGF-induced angiogenesis in mouse retina [65], with loss of signaling resulting in compromised vasculature formation. Both *SEMA3C* and its cognate receptor, *PLXND1*, were expressed at higher levels in the anterior pituitary of HG birds. The VEGF receptor *FLT1* was also expressed at higher levels in HG birds at all ages. The Wnt protein receptor FZD4 has been shown to be necessary for blood vessel morphogenesis [66] and was expressed at higher levels in HG birds at later ages. Elevated *PTPRZ1* mRNA levels in the pituitary gland of HG birds may also contribute to alterations in MDK-PTPRZ1 signaling leading to differences in pituitary gland structure and organization in addition to putative regulation of hormone production, as MDK has been shown to positively influence cell proliferation, cell migration, and angiogenesis [67]. Together, these results indicate that there may be differences between the lines related to connectivity between hypothalamic neurons, the hypophyseal portal vasculature, and the anterior pituitary gland. Alterations in microvascular formation and support may contribute to differences in metabolic feedback to the central neuroendocrine system between the genetic lines, as well as affect delivery of hypothalamic inputs to the pituitary gland or the delivery of anterior pituitary hormones into peripheral circulation.

## Conclusions

While intensive genetic selection within the broiler industry has led to rapid advances in growth rate, body composition (improved muscle accretion accompanied by increases in abdominal fat), and feed efficiency, there is still a lack of understanding related to endocrine control of growth and metabolism in these animals. As animal production systems are forced to reduce the use of antibiotics due to consumer demand and regulatory requirements, understanding biological mechanisms governing growth and metabolism in broiler chickens is crucial to maintaining production efficiency. In this study, transcriptional profiling followed by several bioinformatics approaches were used to evaluate gene expression within anterior pituitary glands of juvenile male broiler chickens selected for high or low body weight in order to identify endocrine mechanisms regulating phenotypic differences between these animals. A total of 263 candidate genes with a potential role in altering growth and metabolism were identified, and the results clearly demonstrate that molecular events associated with all levels of pituitary gland function were impacted by divergent selection. Among the candidate genes were five of the six pituitary hormones. Organization of the dataset using SOMs identified clusters of genes which could be driving phenotypic differences through altered regulation of pituitary hormone production. GO categorization and pathway analyses confirmed that processes and gene networks related to signal transduction, transcriptional regulation, and membrane trafficking and vesicle-mediated transport were different between lines and may be influencing hormone expression and secretion. Differential expression of genes involved in immune regulation was observed, suggesting that processes such as inflammation and response to cellular stressors may compromise pituitary function. Finally, genes playing a role in processes related to morphology and angiogenesis were highlighted, providing evidence that pituitary function is intimately tied to structure and that pituitary gland organization will influence hypothalamic and systemic metabolic inputs and delivery of hormones regulating growth and metabolism into peripheral circulation.

## Methods

### Animals and sample collection

Meat-type chickens maintained at INRA (Nouzilly, France) that have been divergently selected for high or low juvenile body weight at 8 weeks of age were used in this study [6]. Samples used in this study were collected from male birds that were reared as previously reported [15]. Briefly, birds were raised in floor pens (4.4 m × 3.9 m) according to standard broiler practice and given ad libitum access to water and conventional starter (weeks 0–3; 22% crude protein and 3050 kcal ME/kg) or grower (weeks 3–11; 20% crude protein, and 3100 kcal) rations. To reduce early mortality of LG birds and minimize environmental differences, the two lines were raised separately through 3 weeks of age and then placed together and raised as a mixed population for the remainder of the study. At post-hatch weeks 1, 3, 5, and 7, randomly selected birds from each group (*n* = 8 per line) were weighed, and blood was collected from the brachial vein using heparinized syringes for plasma isolation. Following cervical dislocation, abdominal fat pad weights were determined, and anterior pituitary glands were collected, immediately snap-frozen in liquid nitrogen, and stored at -80 °C until RNA extraction. Plasma levels of total T_3_ and T_4_ were determined using commercially available coated tube radioimmunoassay kits (MP Biomedicals, Solon, OH). All procedures were approved by the Institutional Animal Care and Use Committee at each of the four institutions (INRA, University of Maryland, University of Delaware, and University of Georgia).

### RNA isolation and amplification

Anterior pituitary total RNA was isolated from four birds of both lines at each of four ages (1, 3, 5, and 7 weeks post-hatch; *n* = 4) using the RNeasy Mini Kit (Qiagen, Valencia, CA) with on-column DNase digestion to eliminate potential genomic DNA contamination. Isolated total RNA was quantified with a spectrophotometer by measuring absorbance at 260 nm, and the quality was verified with a bioanalyzer (Agilent Technologies, Palo Alto, CA). As chicken anterior pituitary glands do not yield sufficient total RNA for direct analysis by microarray, a previously detailed and validated [68–70] modification of the Eberwine procedure [71] was used to amplify mRNA for hybridization. Briefly, total RNA (0.5 μg) was reverse transcribed with SuperScript II (Invitrogen, Carlsbad, CA) using an oligo (dT) primer containing a T7 promoter site (5′-GGCCAGTGAATTGTAATACGACTCACTATAGGGAGGCGGT_24_–3′; Affymetrix, Santa Clara, CA). After second-strand synthesis, the double-stranded cDNA was phenol-chloroform extracted, purified using a Microcon-30 column (Millipore, Billerica, MA), and used as a template for in vitro transcription with the T7 MEGAscript Kit (Ambion, Austin, TX) according to the manufacturer’s protocol. The resulting amplified RNA (aRNA) was phenol-chloroform extracted, purified with a Spin Column-30 (Sigma, St. Louis, MO), and quantified using the RiboGreen RNA Quantitation Kit (Invitrogen).

### Microarray hybridization, processing, and analysis

The Del-Mar 14 K Chicken Integrated Systems Microarray (Geo Platform accession no. GPL1731), an annotated cDNA array that has been previously described [24, 72] was used for transcriptional profiling of anterior pituitary mRNA expression in HG and LG chickens. A total of 32 individual animals were analyzed, with four replicate samples (*n* = 4) from each of the two lines (HG and LG) at each of the four ages (weeks 1, 3, 5, and 7). A reference hybridization design [73] was used for microarray analysis, where an internal reference pool was generated from equal amounts of aRNA from each sample. Individual aRNA samples from each animal were labeled with Cy3, an aliquot of the reference sample was labeled with Cy5, and equal amounts of each (1 μg) were hybridized together on a slide. This design necessitated the use of 32 microarray slides (one per individual pituitary gland). Labeling with Cy3 and Cy5, microarray hybridization, and image scanning were performed at the University of Maryland Biotechnology Institute’s Microarray Core Facility as previously described [68, 69].

Data from the microarray analysis were processed, normalized, and trimmed as described earlier [28, 68, 69] using software that is part of the TM4 suite of microarray data analysis applications [74] freely available from The Institute for Genomic Research (TIGR, Rockville, MD). During image processing, cDNA spots that were flagged due to lack of detection, detection below background, pixel intensity saturation, or malformation were rejected from further processing and normalization and removed from the dataset. Spots whose pixel intensity for all slides was < 90% of the lowest median pixel intensity for the salmon DNA control spots (background; 8 spots/slide) among all slides were also eliminated from further consideration, as were any spots that were not detectable on at least half of the slides. Microarray data files for the study were deposited in the GEO data repository (accession no. GSE122519, sample accession nos. GSM3473153 – GSM3473183).

The trimmed dataset consisted of 10,437 cDNA spots that were submitted for statistical analysis by two-way analysis of variance (ANOVA) using the general linear model (GLM) procedure of Statistical Analysis System (SAS Institute, Cary, NC) v. 9.4. Data were analyzed as log_2_(normalized Cy3/raw Cy5), or log_2_-ratio, for each spot, and differences were considered statistically significant at *P* ≤ 0.05. Genes with significant differences for the line-by-age interaction, main effect of line, and main effect of age are listed in Additional file 2. To further filter out false positives, these 2310 spots were considered identified as DEGs if the fold-difference among experimental groups was ≥1.6 (0.68 on a log_2_ scale). This resulted in 291 DEGs of interest for the line-by-age interaction or main effect of line, which were further subjected to downstream analysis for GO categorization, SOMs clustering, and IPA gene network and upstream regulator analysis.

For purposes of comparison between microarray expression data and RT-qPCR expression data (see below), data were transformed as 2^(log2ratio)^ and divided by the mean value of the age and line with the highest expression for that spot so that data are expressed relative to the age and line with the highest expression level (set to 100%). Relative expression data for individual values were log_2_-transformed prior to analysis using two-way ANOVA, and differences between groups were determined with the test of least significant difference (SAS) when the overall *P*-value for a given effect (genetic line-by-age, genetic line, or age) was statistically significant (P ≤ 0.05).

### Gene ontology and self-organizing maps analyses

DEGs were analyzed for GO enrichment terms based on biological process and molecular function using AgBase (http://agbase.arizona.edu/), a resource for functional analysis of agricultural gene products [75]. Of the 260 functionally annotated DEGs, 249 and 236 were successfully placed into GO categories based on biological process and molecular function, respectively. GeneCluster 2.0 [76] was used to organize the 291 DEGs into 16 SOMs clusters in a 4 × 4 configuration. Data were entered into the program as log_2_ratio_HGmean_ – log_2_ratio_LGmean_ at each age, in order to identify changes in relative expression between the lines which may be correlated with divergence in growth and body composition. A 4 × 4 grid was chosen in an iterative process to minimize both the variance within individual clusters and the redundancy of similar clusters, while still maintaining profiles that reflected divergence of HG and LG phenotypes.

### Gene interaction network and upstream regulator identification

The 260 DEGs that were functionally annotated using GeneBase were submitted to IPA in order to identify gene interaction networks and upstream regulators. Of these, 249 DEGs mapped to the IPA annotated database and were subjected to further analysis by the program. Data were entered into IPA as relative expression between HG and LG birds, (log_2_ratio_HGmean_ – log_2_ratio_LGmean_), for each mapped DEG at each of the four ages.

### RT-qPCR analysis

Two-step RT-qPCR was used to determine expression levels of pituitary hormone transcripts, as well as confirm expression patterns of 19 DEGs. Total RNA (1 μg) was used for reverse transcription reactions (20 μl) carried out with SuperScript III (Invitrogen) and an oligo (dT) primer [5′-CGGAATTCTTTTTTTTTTTTTTTTTTTTV-3′; Integrated DNA Technologies (IDT), Coralville, IA]. As a negative control for genomic DNA contamination, a pool of RNA from all samples was made, and the reaction was conducted as the others except the reverse transcriptase was not added (no-RT control). All reactions were diluted to 100 μl (5-fold) before quantitative PCR analysis.

Primers (IDT) used for PCR were designed with Primer Express Software (Applied Biosystems, Foster City, CA) from transcripts annotated in Ensembl chicken genome assembly Galgal4 (http://www.ensembl.org/Gallus_gallus/Info/Index), where possible. Primers for genes with missing or problematic annotations were designed using sequences in GenBank (https://www.ncbi.nlm.nih.gov/genbank/). Primers (Additional file 4) with parameters as described previously [77] were designed to span an intron within the 3′-end of the transcript, whenever possible. PCR reactions (15 μl) contained 1 μl diluted cDNA, 400 nM each primer, PCR buffer (50 mM KCl, 10 mM Tris–HCl, 0.1% triton-X-100), 0.12 U/μl Taq Polymerase, 200 nM dNTPs, 40 nM fluorescein (Invitrogen), and SYBR Green I Nucleic Acid Gel Stain (Invitrogen) diluted 1:10,000 and were carried out in the MyiQ Single-Color Real-Time PCR Detection System (Bio-Rad, Hercules, CA). Each PCR reaction was conducted in duplicate, and the Ct value used in subsequent calculations was the mean of the values from these duplicate reactions. PCR cycling conditions were as follows: initial denaturation at 95 °C for 3 min followed by 40 cycles of 95 °C for 15 s, 60 °C for 30 s, and 72 °C for 30 s. Dissociation curve analysis and gel electrophoresis were conducted to ensure that a single PCR product of appropriate size was amplified in each reaction and was absent from the no RT controls. Levels of mRNA for each candidate gene were normalized to mRNA levels of glyceraldehyde phosphate dehydrogenase (GAPDH). A variation of the comparative Ct method [78] was used to assess relative gene expression as previously described [77]. Briefly, each candidate mRNA was normalized to GAPDH mRNA and results expressed relative to the age and line with the highest expression level (equal to 100%). Data were log_2_-transformed prior to statistical analysis as described above for individual cDNA probes printed on the microarray.

## Additional files

Additional file 1: Significant gene lists. A Microsoft excel file containing three worksheets listing all genes that had a statistically significant (*P* ≤ 0.05) line-by-age interaction (“Line-by-Age Interaction”), main effect of line (“Main Effect of Line”), or main effect of age (“Main Effect of Age”). Each list contains the clone ID, the clone GenBank accession number, the clone BlastX and BlastN hits, *P*-value, Log_2_-fold difference between high growth and low growth lines, and the mean value for each line at each age. Genes highlighted in blue on each sheet have a greater than 1.6-fold difference in expression between the highest value in one line and the lowest value in the other. (XLSX 675 kb)

Additional file 2: Differentially expressed genes (DEGs) and self-organizing maps (SOMs) clusters. A Microsoft excel file containing a single worksheet (“Differentially Expressed Genes”) listing all DEGs that had at least 16 observations, a statistically significant (P ≤ 0.05) line-by-age interaction or main effect of line, and at least a 1.6-fold difference between the lines. For each gene, the SOMs cluster, platform ID, clone ID, clone GenBank accession number, clone name, gene symbol, human protein ID, *P*-values for the line-by-age interaction or main effect of line, and log_2_ratio for each age are provided. (XLSX 62 kb)

Additional file 3: Biological functions. A Microsoft excel file containing two worksheets listing the top five molecular and cellular function (“Mol and Cell Func”) and physiological system development and function (“Physiol Sys Dev and Func”) categories as determined by Ingenuity Pathway Analysis. Each sheet lists the category, functional annotation, P-values for overrepresentation, and a gene list for each functional annotation. (XLSX 18 kb)

Additional file 4: Primer sequences. A Microsoft excel file containing a single worksheet (“RT-qPCR primers”) listing the primers used for RT-qPCR analysis. The gene symbol, full gene name, Ensembl chicken genome assembly Galgal4 transcript ID, forward and reverse primer sequences, and amplicon size are provided for each primer pair. (XLSX 12 kb)

## Abbreviations

5HTR1B: Serotonin receptor 1B
ACTH: Adrenocorticotropic hormone
ADCY2: Adenylate cyclase 2
AKAP7: A-kinase anchoring protein 7
ALVE: Avian leucosis virus envelope
ANKRD27: Ankyrin repeat domain-containing protein 27
ANXA2: Annexin A2
B2M: Beta-2-microglobulin
CALR: Calreticulin
cAMP: Cyclic adenosine monophosphate
CASP8: Caspase 8
CDKN1C: Cyclin dependent kinase inhibitor 1C
CGA: α-glycoprotein hormone subunit
CHGB: Chromogranin B
CLU: Clusterin
COL1A2: Collagen, type I, alpha 2
CPNE1: Copine 1
DDIT4: DNA damage-inducible transcript 4
DEG: Differentially expressed genes
DIO: Deiodinase
EGR1: Early growth response protein 1
ERK1/2: Extracellular signal-regulated kinase 1/2
FLT1: Fms-related tyrosine kinase 1
FSH: Follicle-stimulating hormone
FZD4: Frizzled-4
GH: Growth hormone
GHR: Growth hormone receptor
GO: Gene ontology
GRIA1: Glutamate receptor, ionotropic, AMPA 1
HBA1: Hemoglobin alpha 1
HBA2: Hemoglobin alpha 2
HCK: Hematopoietic cell kinase
HG: High growth
HLA-A: Human leukocyte antigen A
HNRNPA2B1: Heterogeneous nuclear ribonucleoprotein A2/B1
HSPA5: Heat shock protein family A, member 5
HWS: High weight selected
IGF: Insulin-like growth factor
INRA: Institut National de la Recherche Agronomique
IPA: Ingenuity Pathway Analysis
IRF1: Interferon regulatory factor 1
LG: Low growth
LH: Luteinizing hormone
LWS: Low weight selected
MAP3K7: Mitogen-activated protein kinase kinase kinase 7
MAPRE2: Microtubule associated protein RP/EB family member 2
MDK: Midkine
MEK1: Mitogen-activated protein kinase/extracellular signal-regulated kinase kinase 1
MHC: Major histocompatibility complex
NR0B1: Nuclear receptor subfamily 0, group B, member 1
NR3C1: Nuclear receptor subfamily 3, group C, member 1 (glucocorticoid receptor)
OAS3: 2′-5′-oligoadenylate synthetase 3
OASL: 2′-5′-oligoadenylate synthetase like
PLXND1: Plexin D1
PODXL: Podocalyxin-like protein 1
POMC: Pro-opiomelanocortin
PPP3R1: Protein phosphatase 3 regulatory subunit B alpha
PRL: Prolactin
PTPRZ1: Protein tyrosine phosphatase, receptor type Z1
RASD1: Dexamethasone-induced ras-related protein 1
RT-qPCR: Reverse transcription-quantitative PCR
SEMA3C: Semaphorin 3C
SEMA3F: Semaphorin 3F
SMAD1: Mothers against decapentaplegic homolog 1
SOMS: Self-organizing maps
STAT1: Signal transducer and activator of transcription 1
STMN1: Stathmin 1
T_3_: 3,5,3′-triiodothyronine
T_4_: Thyroxine
TCR: T-cell receptor
TSH: Thyroid-stimulating hormone
UNCX: UNC homeobox
VEGF: Vascuar endothelial growth factor
